# In Vivo Confocal Microscopy of Cornea in Patients with Terrien's Marginal Corneal Degeneration

**DOI:** 10.1155/2019/3161843

**Published:** 2019-07-11

**Authors:** Ting Chen, Qiangxiang Li, Xiangbo Tang, Min Liao, Hua Wang

**Affiliations:** ^1^Department of Ophthalmology, Xiangya Hospital, Central South University, Changsha, Hunan Province, China; ^2^Department of Ophthalmology, Xiangya Changde Hospital, Changde, Hunan Province, China; ^3^Hunan Provincial People's Hospital, Changsha, Hunan Province, China

## Abstract

This study was aimed at observing the morphological changes of the cornea with ocular in vivo confocal microscopy (IVCM) in patients with Terrien's marginal degeneration (TMD). Ten patients (20 eyes) with TMD treated in the Department of Ophthalmology, Xiangya Hospital, and 10 healthy controls (20 eyes) were included in the current study. A detailed slit lamp microscopy, anterior segment photography, and corneal IVCM examination were performed for each eye. The density of central and marginal corneal epithelial cells, stromal cells, and subepithelial nerve fibers was compared between the two groups using the Wilcoxon rank sum test. Compared with the control group, the corneal epithelial and endothelial cells in the TMD group showed granular highly reflective substances and thinner subepithelial nerve fibers. The uneven dot-like highly reflective substances without cell structures appeared in the stromal layer of the cornea. The density of central and marginal corneal epithelial cells, stromal cells, and subepithelial nerve fibers was lower in the TMD group (*p* < 0.05), and they were negatively correlated with severity of the disease (*p* < 0.05). Our study demonstrated that the density of corneal epithelial cells, stromal cells, and sensory plexus nerve fibers was significantly reduced in the TMD group. The pathological changes were more obvious in the marginal cornea, and it is correlated with severity of the disease.

## 1. Introduction

Terrien's marginal degeneration (TMD) is a chronic and bilateral marginal keratopathy [1], mainly characterized by sulcus thinning of corneal margins, corneal stromal atrophy, and lipid deposition, often accompanied by corneal neovascularization. It progresses to corneal staphyloma and eventually corneal perforation in its advanced period. The onset age of TMD patients is 10–70 years old, about 2/3 of which are middle-aged men about 40 years old. The duration of the disease can be up to 10–30 years. The lesion in the corneal limbus was mostly seen at 9–3 o'clock positions, and the upper corneal limbus is the most common site for initial lesions, fast development, and later perforations. Although TMD could progress to advanced stages, severe symptoms like red eyes, eye pain, and corneal perforation are rarely found. The main manifestation of this disease is the gradual decrease of visual acuity. With the progression of TMD, the cornea became thinner and expanded. The curvature of the cornea and the converse astigmatism gradually increased, which led to a progressive decline of the patients' visual acuity. The exact pathogenesis remains unknown; however, it is commonly believed that TMD is associated with the following factors: (a) immune diseases, (b) nutritional disorders and degenerative diseases, (c) inflammatory diseases, and (d) abnormality secondary to the composition of tears [2].

At present, the objective examination methods of TMD in the clinic include ultrasound biomicroscopy of the anterior segment, optical coherence tomography (OCT) of the anterior segment, corneal topography, and IVCM examination. Among all the above-mentioned methods, IVCM is the only one that can observe the normal and pathological corneal characteristics at the living corneal cell level. At present, there are only a few reports that IVCM is used to observe TMD. Some studies have found that TMD had hyperreflective substances deposition in the corneal epithelium, abnormal aberration of nerve fibers, activation of stromal cells, no cellular structural substances, and deposition of lipid layer particles by IVCM [3, 4]. However, no previous studies have reported the correlation of IVCM findings and the severity of TMD or compared the pathological changes between the central and marginal cornea.

The clinical diagnosis for some early and atypical cases is difficult, which results in poor therapeutic effect in severe cases. In the present study, we investigated the corneal characteristic pathological changes of TMD on the in vivo level of cells by IVCM, which provides a clinical basis for clarifying the pathogenesis of TMD and meanwhile provides a sensitive and specific detection method for the clinical diagnosis and follow-up of TMD patients.

## 2. Subjects and Methods

### 2.1. Subjects

Patients diagnosed with TMD for the first time from July 2015 to June 2017 in the Department of Ophthalmology, Xiangya Hospital, Central South University, were included in the study. No gender or age restrictions were applied. Patients with history of ocular surgery, other corneal lesions by any causes, systemic diseases, immune system diseases, and psychological or mental illness were excluded from the study. Meanwhile, ten healthy volunteers matched by age and gender with no ocular or systemic diseases were recruited as controls.

### 2.2. Evaluation for Severity of Disease

**Figure 1:**
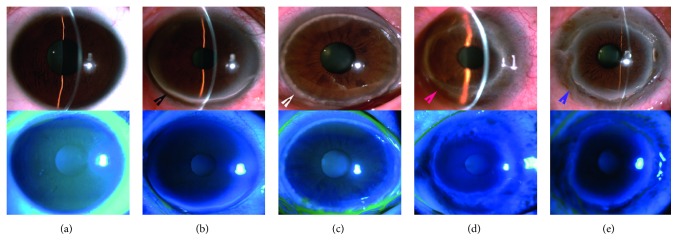
Anterior segment photography of TMD at various stages. (a) Normal eyes; (b) TMD at Stage I: the gray-white turbidity at the corneal margin parallel to the corneal limbus (black arrow); (c) TMD at Stage II: the sulcus shape of the cornea was thinned, and there was lipid deposition in the sulcus (white arrow); (d) TMD at Stage III: the cornea in the lesion zone became thinner to form one or more bulging zones with a width of 1.5–3 mm or above (red arrow); (e) TMD at Stage IV: the marginal cornea was further thinned (blue arrow), with protruded central cornea.

Patients with TMD were classified into Stage I, Stage II, Stage III, and Stage IV by the Francois staging method [5, 6]. Stage I: Infiltration Period: the gray-white turbidity at the corneal margin parallel to the corneal limbus was accompanied with the neovascularization in the superficial cornea. Stage II: Degeneration Period: the sulcus shape of the cornea was thinned, and there was lipid deposition in the sulcus. Stage III: Swelling Period: the cornea in the lesion zone became thinner to form one or more bulging zones with a width of 1.5–3 mm or above, and meanwhile, the significant astigmatism against the rule was found. Stage IV: Keratoconus Period: the lesion protruded forward to form the keratoconus, which may be prone to corneal perforation (Figure 1) [5, 6].

### 2.3. Inspection Methods

#### 2.3.1. Slit Lamp Microscopy (Keeler Optics)

The anterior segment was checked using a slit lamp.

#### 2.3.2. Anterior Segment Photography (Topson BG4)

The corneal photos were taken before and after corneal fluorescein staining.

#### 2.3.3. Corneal IVCM Examination

An in vivo laser confocal microscope (Heidelberg Engineering, Laser class1) was used to perform IVCM. For each eye, different layers of corneal cells in both the central and peripheral cornea as well as the corneal subepithelial sensory plexus were scanned. All examinations were finished by one skilled physician with the same machine. The most affected portion of the cornea in all patients was examined.

### 2.4. Measurement of Cell Density

The random number method was used to select 3 frames of pictures randomly at each level, each frame of picture was divided into 36 equal parts on average, and the random number method was used to select 10 equal parts randomly. The average corneal cell density was calculated using the built-in cell counting software [7]. The principle of cell counting was as follows: only the complete number of cells; when the cell is pressed on the grid line, only count the upper line, not the lower line, only the right line, not the left line.

### 2.5. Measurement of the Density of Subepithelial Nerve Fibers

At least 3 images of subepithelial nerve fibers were randomly selected for analysis in each case. The density of nerve fibers (the total length of nerve fibers in a picture) was measured by semiautomatic neural analysis and the tracking system (Neuron J) [8]. The data obtained from Neuron J were converted into the corneal subepithelial nerve density (*μ*m/mm^2^) according to the image size and pixel conversion unit. Because the size of each image is 384 pixels × 384 pixels, the actual size is 400 *μ*m × 400 *μ*m, so it is 1 *μ*m = 0.96 pixels. Therefore, the conversion formula is as follows: corneal subcutaneous nerve density (*μ*m/mm^2^) = total length of nerve fiber [*μ*m]/4002 × 1,000,000 = total length of nerve fiber [pix]/0.96 × 6.25 [9].

### 2.6. Statistical Analysis Method

The experiment results underwent a descriptive statistical analysis based on the software SPSS 22.0 (IBM, Armonk, NY). After the normality test, the data of two groups showed a skewed distribution. Therefore, the median (median, *M*) ± quartile spacing (quartile, *Q*) was taken to describe the central location and dispersion of the statistical data. The Wilcoxon rank sum test was used to compare the density of central and marginal corneal cells and subepithelial nerve fibers between the TMD group and control group. It is also used to compare the density of corneal cells and subepithelial nerve fibers in the central and marginal cornea in the TMD group. The Spearman rank correlation method was used to analyze the correlation between the density of corneal cells and subepithelial nerve fibers and the severity of the disease. *p* < 0.05 was considered to have statistical significance.

## 3. Results

### 3.1. General Characteristics

There were six TMD female patients and four TMD male patients, with a mean age of 51.9 ± 20.3 years. Among them, eight patients had binocular disease, accounting for 80% of the group. There were three patients (5 eyes) with TMD in the Stage-I period, three patients (5 eyes) with TMD in the Stage-II period, two patients (4 eyes) with TMD in the Stage-III period, and two patients (4 eyes) with TMD in the Stage-IV period (Figure 1). The control group included 4 male and 6 female, aged 17–75 years, with a mean age of 49.0 ± 24.3 years.

### 3.2. Change of Corneal Cell Morphology in TMD Patients by IVCM

#### 3.2.1. Epithelial Layer

**Figure 2:**
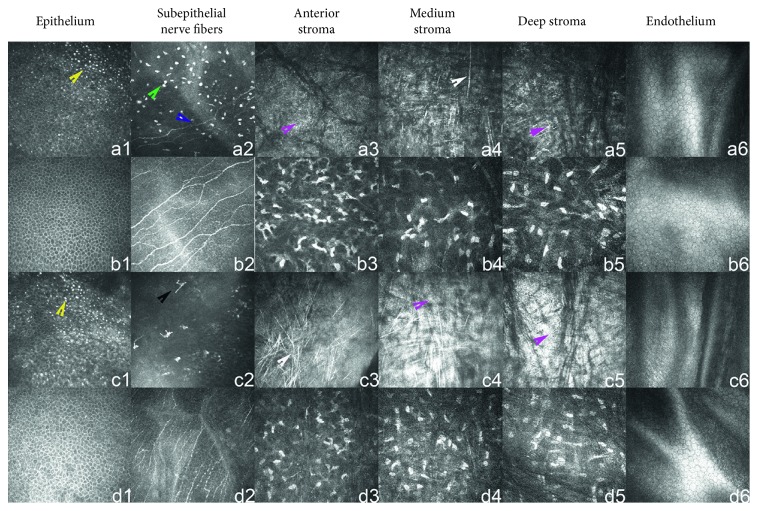
Corneal structures under IVCM. a1–a6 are the central corneal structures of TMD patients, b1–b6 are the central corneal structures of the controls, c1–c6 are the marginal corneal structures of TMD patients, and d1–d6 are the marginal corneal structures of the controls. The density of epithelial cells in the central and marginal cornea of TMD patients was decreased, the cell boundaries were blurred, and the dot-like highly reflective substances appeared; the density of the subepithelial nerve fiber plexus was significantly reduced and even disappeared (blue arrow), while the density of inactivated Langerhans cells (green arrow) and activated Langerhans cells (black arrow) was increased. The number of anterior, medium, and deep stromal cells of the cornea was decreased, the dot-like (yellow arrow), needle-like (white arrow), and flaky highly reflective substances without cell structures (red arrow) were presented, and the boundaries of endothelial cells were blurred.

The corneal epithelial layer in the marginal and central cornea of both eyes in 10 TMD patients showed a decrease in cell density (*p* < 0.05) compared with controls. They also displayed a blurred boundary and deposition of dot-like and massive highly reflective substances. The lesion was more severe in the marginal cornea compared with the central cornea (*p* < 0.05) (Figure 2).

#### 3.2.2. Subepithelial Nerve Plexus

The affected eyes of 10 TMD patients showed a significant decrease in the density of subepithelial nerve fibers (*p* < 0.05) compared with controls. We could see a bent nerve trend, thinned nerve fibers, and even an absence of subepithelial nerve fibers in severe patients. The activation of dendritic cells was found in the nerve fiber layer of some patients, and dot-like, massive, or cord-like lipid-like particles were found locally. The lesion in the corneal marginal zone was more severe than that in the corneal central zone (*p* < 0.05) (Figure 2).

#### 3.2.3. Corneal Stroma

The corneal stroma of TMD patients showed a variety of morphological changes by IVCM, including stromal neovascularization, bright cell nuclei, enhanced cytoplasmic reflex, activated stromal cells, a decrease or absence of stromal cells, dot-like, needle-like, highly reflexive, and uneven fiber tissue without cell structures. Besides, the morphological change was more significant in the marginal corneal stromal zone than that in the central corneal zone (*p* < 0.05) (Figure 2).

#### 3.2.4. Endothelial Layer

The corneal endothelial cells of 11 eyes in TMD patients showed dot-like reflections and blurred boundaries of endothelial cells, accounting for 55% of all cases (Figure 2).

#### 3.2.5. Langerhans Cells

A large number of dendritic activated Langerhans cells were found in the Bowman membrane and subepithelial nerve plexus of two TMD patients (4 eyes), accounting for 20% of all cases. A large number of inactivated Langerhans cells without dendritic were found in the Bowman membrane and subepithelial nerve plexus of one TMD patient (1 eye), accounting for 5% of all cases (Figure 2).

### 3.3. Density of Corneal Cells and Density of Subepithelial Nerve Fibers

The density of central and marginal corneal epithelial cells; superficial, medium, and deep stromal cells; and subepithelial nerve fibers in patients of TMD group was significantly lower than that of the control group, and the difference was statistically significant (*p* < 0.05); no significant difference was found in the density of endothelial cells between the two groups (*p* > 0.05). In the TMD group, the density of epithelial cells; anterior, medium, and deep stromal cells; and subepithelial nerve fibers in the central cornea was significantly higher than that in the marginal cornea, and the difference was statistically significant (*p* < 0.05). However, no significant difference was found in the density of endothelial cells between the two groups (*p* > 0.05) (Tables 1–4). The density of corneal epithelial cells; superficial, medium, and deep stromal cells; and subepithelial nerve fibers in TMD patients was negatively corrected with the severity of the disease (Table 5).

## 4. Discussion

Marginal corneal degeneration, also known as Terrien's corneal degeneration (TMD), was first reported by Trumpy in 1881 as “corneal hyaline degeneration” and described by Terrien in 1990. Suveges and associates demonstrated TMD as a disease of asymmetric progression in both eyes, while Austin and Brown classified TMD into quiescent period and advanced period through the histological presence of inflammation in TMD patients. Moreover, many researchers believed that inflammation played a key role in the progression of TMD and the inflammatory cells could promote the progression of TMD. TMD has been recognized to a certain extent, but it is still restricted to understand the tissue and cytological features of TMD. TMD presents no specific changes in pathological morphology, and it is mainly characterized by sulcus thinning of noninflammatory corneal stroma in the lesion zone, accompanied by superficial neovascularization and lipid deposition. With integral corneal epithelium, the epithelial cells can proliferate to more than 10 layers, accompanied by abnormal proliferation of basal membrane tissues. Historically, the cytologic examination of TMD could only rely on the pathologic and other invasive examination methods. The invention and application of IVCM made it possible to perform the noninvasive biopsy of corneal conjunctival lesions at the cellular level. However, there are still few studies on the characteristic pathological changes of TMD using IVCM.

IVCM is an instrument equipped with the laser scanning device based on the ordinary microscope imaging, and it uses the laser to stimulate the fluorescence probe to perform the in vivo noninvasive examination and obtain the fluorescence images of cells or cell structures within tissues through image processing of the computer. IVCM has been widely applied in many fields, such as cell biology, microbiology, and neurobiology, and its application field has gradually expanded from the basic disciplines to the clinical applications. IVCM is also getting popularity in ophthalmology, and the IVCM can be used in almost all lesions involving the ocular surface to observe the microstructure change of the ocular surface resulted from the lesions. The concept of confocal microscopy was first patented by Marvin Minsky in 1957 to study the brain neural cells [10]. The first in vivo images of the human cornea were obtained by Cavanaugh et al. in 1989. They demonstrated the confocal visualization of the epithelium, basal lamina, Bowman layer, stromal nerves, pre-Descemet membrane, and endothelium in the living cornea. In 1994, James Hill invented the tandem ocular scanning device, IVCM. Thus far, three main commercial confocal systems have been developed for in vivo corneal imaging: the tandem scanning confocal microscope (TSCM), the slit scanning confocal microscope (SSCM), and the laser scanning confocal microscope (LSCM) [11, 12]. At present, IVCM has been widely applied to the studies of corneal tissues and keratonosus. The scope of studies now covers the studies on normal human cornea, contact lens wear, keratoconus, dry eye disease, neurotrophic keratopathy and infectious keratitis, corneal dystrophies, epithelial and subepithelial corneal dystrophy, stromal dystrophies, endothelial dystrophies, other ocular diseases, neuropathic corneal pain, penetrating keratoplasty, phototherapeutic keratectomy, photorefractive keratectomy, laser-assisted in situ keratomileusis, laser-assisted subepithelial keratectomy, autoimmune diseases, peripheral neuropathies, diabetes mellitus, and other systemic diseases [13–26]. IVCM has been widely used in the study of keratoconjunctivitis, but so far, there are only few reports on the study of using IVCM to observe the characteristic corneal lesions of TMD patients, and there is still no comparative analysis study on marginal and central corneal lesions in TMD patients.

Ferrari's IVCM examination of a patient with early TMD showed the highly reflective substances in the lesion of the patient, the activated anterior stroma for honeycomb changes, the decreased density of subepithelial nerve plexus, and the abnormality of the epithelial basement membrane and Bowman membrane. Meanwhile, Ferrari et al. also found the abnormality of the subepithelial nerve plexus and the activated anterior stromal cells and dendritic cells in the contralateral eye of the patient [3]. The above findings were also reported later by Ceresara et al., MD, in their observation of right-eye TMD patient by IVCM. Ceresara et al., MD, noted the infiltration of inflammatory cells, absence of the epithelial basement membrane, and neovascularization in the lesion zone of the right-eye TMD patient under IVCM [4]. In the current study, the density of corneal epithelial cells in the TMD patients was also found to be reduced, which was correlated with the severity of the disease. Moreover, the boundary of corneal epithelial cells was blurred, accompanied by dot-like, massive, and cord-like highly reflective substance deposition, and the corneal degeneration of the marginal zone was more severe than that of the central zone.

In this study, we also found a decreased density of marginal and central corneal epithelial nerve fibers in both eyes of all TMD patents, the broken subepithelial nerve fibers, the absence of subepithelial nerve fibers for severe patients, the bent nerve trend, the thinned nerve fibers, activated dendritic cells in nerve fiber layers of some patients, the increased density, and dot-like, massive, and cord-like highly reflective substances. Moreover, the density reduction of subepithelial nerve fiber layer was positively corrected with the severity of TMD. And the corneal degeneration of the marginal zone was more severe than that of the central zone in all patients. The decrease in the density of the subepithelial nerve fiber plexus is found in many diseases, and our study has further demonstrated that TMD can also be involved in the corneal subepithelial nerve fibers.

TMD is a corneal stromal atrophy disease. Our study further confirmed the gradual decrease of corneal stromal cells of TMD patients and the loss of normal stromal structure with the development of the disease. We also found that the anterior stromal cells in TMD patients were not necessarily in an activated state, while they were associated with the severity and location of the disease. The central and corneal anterior stromal cells in TMD patients at Stages I and II could be activated, and the activated anterior stromal cells were mainly located in the central cornea. However, the central and marginal anterior stromal cells in TMD patients at Stages III and IV mainly showed a blurred or fuzzy structure of stromal cells, and even the dot-like, needle-like, and uneven reflections without cell structures were presented. We speculated that the anterior stromal cells in the early TMD period showed an activated state, while, with the development of the disease, the density of stromal cells gradually decreased and disappeared eventually, showing the dot-like, needle-like, and flaky uneven reflections without cell structures. Meanwhile, the similar changes were found in the medium and deep stromal cells.

Our studies showed the dot-like reflections in the corneal endothelium layer, blurred boundary of corneal endothelial cells, and uneven highly reflective deposition in 11 eyes among all 20 eyes. We infer that the corneal degeneration of the TMD patients can also be involved in the corneal endothelium. In previous IVCM studies, the abnormality of the corneal endothelium was not found in TMD patients. This is maybe because there is no comparative analysis of corneal endothelial cells in TMD patients with different pathological changes due to a small number of study cases.

Previous studies showed that TMD was an immune-related disease, and the activated anterior stromal cells and dendritic cells were found in the IVCM examination of TMD patients [3]. Our study also found a large number of activated Langerhans cells in the Bowman membrane and subepithelial nerve plexus of two TMD patients (4 eyes), accounting for 20% of all cases; and a large number of unactivated Langerhans cells in the Bowman membrane and subepithelial nerve plexus of one TMD patient (1 eye), accounting for 5% of all cases. Langerhans cells are immune cells, and a large number of activated Langerhans cells are found in rodent corneal ulcer and other immune-related corneal diseases. However, in this study, they were found only in the Bowman membrane and subepithelial nerve plexus of two TMD patients, suggesting that TMD may be associated with the immunity. However, further studies are needed to observe the existence of other pathogenesis for TMD.

We hypothesized that TMD was a degenerative disease involving all the layers of cornea and the pathogenesis may be associated with the immunity, but there may be other pathogenesis mechanisms which need to be further studied. The degeneration was the first to be involved in the corneal stroma; TMD was a commonly degenerative disease for the marginal and peripheral cornea, and the degeneration of the marginal cornea was earlier and more severe than that of the central cornea. Apart from the corneal endothelial cells in TMD patients, the decrease in the density of corneal stromal cells and the density of subepithelial nerve fibers was negatively corrected with the severity of the disease. As a noninvasive examination tool to observe characteristics of corneal degeneration, IVCM will be of great significance for the diagnosis and follow-up of the TMD patients.

## Figures and Tables

**Table 1:** Comparison of the density of central corneal cells between Terrien's marginal degeneration patients and the control group (*M* ± *Q*).

Group	Case	Density of central corneal cells				
		Epithelium	Anterior stroma	Medium stroma	Deep stroma	Endothelium
TMD group	10	5068.0 ± 1185.0	477.5 ± 744.0	250.0 ± 510.0	250.0 ± 529.0	2686.5 ± 314.0
Control group	10	5930.0 ± 924.0	688.0 ± 519.0	521.5 ± 476.0	484.0 ± 465.0	2864.0 ± 422.0
*z* value	—	−11.436	−4.962	−6.889	−4.072	−1.876
*p* value	—	<0.001^*∗*^	<0.001^*∗*^	<0.001^*∗*^	<0.001^*∗*^	0.061

^*∗*^ *p* < 0.05; TMD = Terrien's marginal degeneration.

**Table 2:** Comparison of the density of marginal corneal cells between Terrien's marginal degeneration patients and the control group (*M* ± *Q*).

Group	Case	Density of marginal corneal cells				
		Epithelium	Anterior stroma	Medium stroma	Deep stroma	Endothelium
TMD group	10	4679.0 ± 2662.0	0.0 ± 0.0	0.0 ± 0.0	0.0 ± 0.0	2765.0 ± 435.0
Control group	10	6139.0 ± 1082.0	412.0 ± 693.0	229.0 ± 606.0	216.0 ± 509.0	2814.0 ± 366.0
*z* value	—	−13.740	−9.948	−6.632	−7.569	−1.568
*p* value	—	<0.001^*∗*^	<0.001^*∗*^	<0.001^*∗*^	<0.001^*∗*^	0.117

^*∗*^ *p* < 0.05; TMD = Terrien's marginal degeneration.

**Table 3:** Comparison of the density of corneal cells between the central corneal zone and marginal corneal zone in Terrien's marginal degeneration patients (*M* ± *Q*).

Group	Case	Density of corneal cells				
		Epithelium	Anterior stroma	Medium stroma	Deep stroma	Endothelium
Central cornea	10	5068.0 ± 1185.3	477.5 ± 744.0	250.0 ± 510.0	250.0 ± 529.0	2708.0 ± 319.0
Marginal cornea	10	4679.0 ± 2001.5	0.0 ± 0.0	0.0 ± 0.0	0.0 ± 0.0	2655.5 ± 362.3
*z* value	—	−3.672	−10.991	−7.407	−9.681	−0.289
*p* value	—	<0.001^*∗*^	<0.001^*∗*^	<0.001^*∗*^	<0.001^*∗*^	0.773

^*∗*^ *p* < 0.05; TMD = Terrien's marginal degeneration.

**Table 4:** Density of subepithelial nerve fibers (*μ*m/mm^2^*M* ± *Q*).

Group	Case	Marginal cornea	Group	Case	Central cornea	Group	Case	Subepithelial nerve fibers
TMD group	10	0.0 ± 0.0	TMD group	10	2082.7 ± 10174.0	TMD marginal cornea	10	0.0 ± 0.0
Control group	10	11555.0 ± 7800.6	Control group	10	12657.6 ± 6657.4	TMD central cornea	10	2082.7 ± 10174.0
*z* value	—	−6.203	—	—	−4.301	—	—	−3.765
*p* value	—	<0.001^*∗*^	—	—	<0.001^*∗*^	—	—	<0.001^*∗*^

^*∗*^ *p* < 0.05; TMD = Terrien's marginal degeneration.

**Table 5:** Correlation analysis of the density of corneal cells and epithelial nerve fibers in patients at stages I, II, III, and IV (*M* ± *Q*).

Staging	Case	Density of corneal cells and epithelial nerve fibers					
		Epithelium	Anterior stroma	Medium stroma	Deep stroma	Endothelium	Subepithelial nerve fibers
Stage I	3	5329.0 ± 1025.0	258.5 ± 756.0	257.5 ± 529.0	250.0 ± 529.0	2819.0 ± 349.0	8045.9 ± 14435.1
Stage II	3	4847.5 ± 1275.0	0.0 ± 274.0	0.0 ± 253.0	0.0 ± 0.0	2654.0 ± 544.0	0.0 ± 1427.7
Stage III	2	4611.5 ± 1341.0	0.0 ± 231.0	0.0 ± 250.0	0.0 ± 0.0	2462.0 ± 643.0	0.0 ± 4707.5
Stage IV	2	0.0 ± 4020.0	0.0 ± 0.0	0.0 ± 0.0	0.0 ± 0.0	2645.0 ± 323.0	0.0 ± 0.0
*r* value	—	−0.499	−0.353	−0.395	−0.190	−0.418	−0.397
*p* value	—	<0.001^*∗*^	<0.001^*∗*^	<0.001^*∗*^	<0.001^*∗*^	>0.99	0.020^*∗*^

^*∗*^ *p* < 0.05.

## Data Availability

The data are available from Ting Chen to researchers who meet the criteria for access to the confidential data.
